# Role of plasma process gas on permeate flux augmentation of cellulose nitrate membrane for mud water treatment

**DOI:** 10.1038/s41598-024-56948-9

**Published:** 2024-03-19

**Authors:** Tonmoi Hazarika, Bharat Kakati, Dipankar Pal, Rimlee Saikia, Ankit Rawal, Manoj Kumar Mahanta, Subir Biswas

**Affiliations:** 1 META Laboratory, Assam Science and Technology University, Jalukbari, Guwahati, Assam 781013 India; 2 Department of Physics, Gauhati University, Jalukbari, Guwahati, Assam 781014 India; 3 Surface Engineering and Plasma Processing Laboratory, Indian Institute of Petroleum and Energy, Visakhapatnam, Andhra Pradesh 530003 India; 4 Pragjyotish College, Pragjyotish Path, Santipur, Guwahati, Assam 781009 India; 5 Physical Science Division, Institute of Advanced Study in Science and Technology, Vigyan Path, Garchuk, Paschim Boragaon, Guwahati, Assam 781035 India

**Keywords:** Cellulose nitrate membrane, Low-pressure plasma, Hydrophilicity, Antifouling, Ageing, Diffusion, Materials science, Physics

## Abstract

A comparative study between Nitrogen (N_2_) and Argon (Ar) plasma is carried out to investigate its effect on surface morphology, hydrophilicity, permeate flux and ageing of cellulose nitrate polymeric membranes in the present work. Langmuir probe and Optical Emission Spectroscopy are used to characterize the plasma. The SEM analysis reveals the noticeable macro-void creations and pore enlargement for both N_2_ and Ar plasma. The AFM analysis shows a higher surface roughness for Ar plasma treatment as compared to N_2_ plasma treatment. XPS analysis confirms the changes in the polymer matrix along with the incorporation of various functional groups on the membrane surface as a result of the plasma treatment. A better hydrophilic nature with prolonged plasma treatment is observed for Ar plasma as compared to N_2_ plasma treatment. The present results show a higher permeate flux with a high rejection rate for Ar plasma treatment in comparison to N_2_ plasma, which might be due to the pore size and pore area enlargement of the membrane. The hydrophobic recovery for both the plasma-treated membranes is found significant for the initial ageing period of 7 days and found almost stable in nature after 7 days. A diffusion-based theoretical model is developed to study the hydrophobic recovery of plasma-treated membranes. A strong alignment between experimental and theoretical results is observed in the present work. The Cake Filtration model, derived from the Hermia model, is identified as the most suitable model for describing the fouling mechanisms for the present work.

## Introduction

Polymeric membranes with a hydrophilic nature have drawn attention of various researchers for the last few decades^1–3^. Most of the polymeric membranes are hydrophobic in nature. Hence, the performance of the membranes depreciates as a result of fouling. Membrane fouling is a common phenomenon for hydrophobic membrane surfaces. This is due to interactions between the solute and the membrane material^4,5^. The separation and permeation characteristics of the membrane are highly dependent on the chemical and physical properties of the polymeric surface^4,6–8^. Thus, hydrophilic surface modification of the membrane surface is a charming approach to deal with the fouling problem.

Several approaches, such as ultraviolet irradiation^9^, plasma treatment^2,10^, gamma irradiation^11^, and chemical reaction^12,13^, have been adopted by different researchers to modify the membrane surface. Among them, plasma-assisted surface modification is an immensely popular method to improve the surface properties of a polymeric membrane, such as adhesion, polarity, and wettability^4,14^. The surface activation of the topmost surface of the polymeric membrane takes place as a result of hydrogen abstraction and radical formation during the plasma treatment^4,11^. Hence, it has the upper hand on selectivity for a particular application without affecting the bulk properties of the polymer^15^. The plasma process gas and its chemical nature play a vital role in the reaction mechanism of plasma surface modification. Several gases like Oxygen^14^, Argon^16^, Helium^17^, Nitrogen^18^, Ammonia^19^, Carbon dioxide^20^, Water^21^, etc. are used as process gas for plasma surface modification. The hydrophilicity induced in the polymeric membrane by integration of the polar function group at the time of plasma treatment can be varied with time. The alteration resulting in decreased hydrophilicity or increased hydrophobicity is referred to as “hydrophobic recovery” or "ageing", representing a prominent drawback of plasma-induced surface modification. This phenomenon is attributed to various physical and chemical processes, which are given below;

1. Re-arrangement of Chemical Groups: This occurs on the surface exposed to plasma treatment due to the conformational mobility of polymer chains.
2. Oxidation and Degradation Reactions: These reactions take place on the surfaces treated with plasma, causing changes in chemical composition and properties.
3. Diffusion of Low Molecular Weight Species: Low molecular weight species from the outer layers diffused into the bulk of the polymer.
4. Plasma-Treatment-Induced Diffusion of Additives: Additives introduced into the polymer from its bulk are diffused towards its surface due to plasma treatment^22^.

The investigation into the effects of plasma treatment on different types of the membranes such as polyacrylonitrile^23^, cellulose nitrate^24^, polyethersulfone^25^, polyacrylonitrile copolymer^26^, polysulfone^27^, polypropylene^28^, regenerated cellulose^29^, cellulose acetate composite polyion complex^30^, polycarbonate^31^, Aromatic Polyamide^32^, polytetrafluoroethylene^33^ has been reported by different researchers. The study reveals the distinct surface modifications as a result of the incorporation of functional groups into the plasma-treated membrane, which alter the antifouling and permeability properties of the membrane. The above investigations manifest the substantial enhancements in wettability, chemical inertness, and permeability for various polymeric membranes subsequent to cold plasma treatment^4,21,23,24,34–39^. The assessment of water contact angle (CA) serves as a pivotal metric for evaluating surface hydrophilicity, contingent upon surface properties like energy and roughness. In the cold plasma process, achieving the lowest water contact angle and maximal adhesion is contingent upon optimizing plasma operating conditions, including treatment time, discharge power, and process gas composition^24,40^.

Cellulose-based materials are widely recognized for their biocompatibility, eco-friendly attributes, non-toxic characteristics, and economically feasible^41^. The global focus on green and sustainable strategies, particularly in the domains of energy and advanced materials, has propelled the importance of such materials. Concurrently, the escalating challenges posed by industrialization have led to a concerning deterioration of water environments, with a pronounced increase in surface water pollution^42^. Thus, it is essential to develop an affordable, safe, and biocompatible cellulose-based membrane with better hydrophilicity and permeation properties for waste-water treatment.

There is a lack of systematic study on the plasma treatment of cellulose nitrate membrane for waste-water treatment applications. In the present work, a comparative study is conducted to assess the influence of Nitrogen and Argon plasma on the surface morphology, wettability, and surface chemistry of cellulose nitrate membranes. Special attention is dedicated to understanding the wettability and ageing behaviour of plasma-treated membranes due to their critical impact. To validate the experimental results, a diffusion-based theoretical model is developed in the present study. A gravity filtration setup is used to explore the antifouling performance of both treated and untreated membranes for both Ar and N_2_ plasma. No literature has been found on studying the fouling mechanism in the gravity filtration process. Additionally, the most suitable model for describing the fouling mechanisms in the present work is also identified using the Hermia model.

## Results and discussion

### Role of plasma process gas on hydrophilicity of the membrane

**Figure 1:**
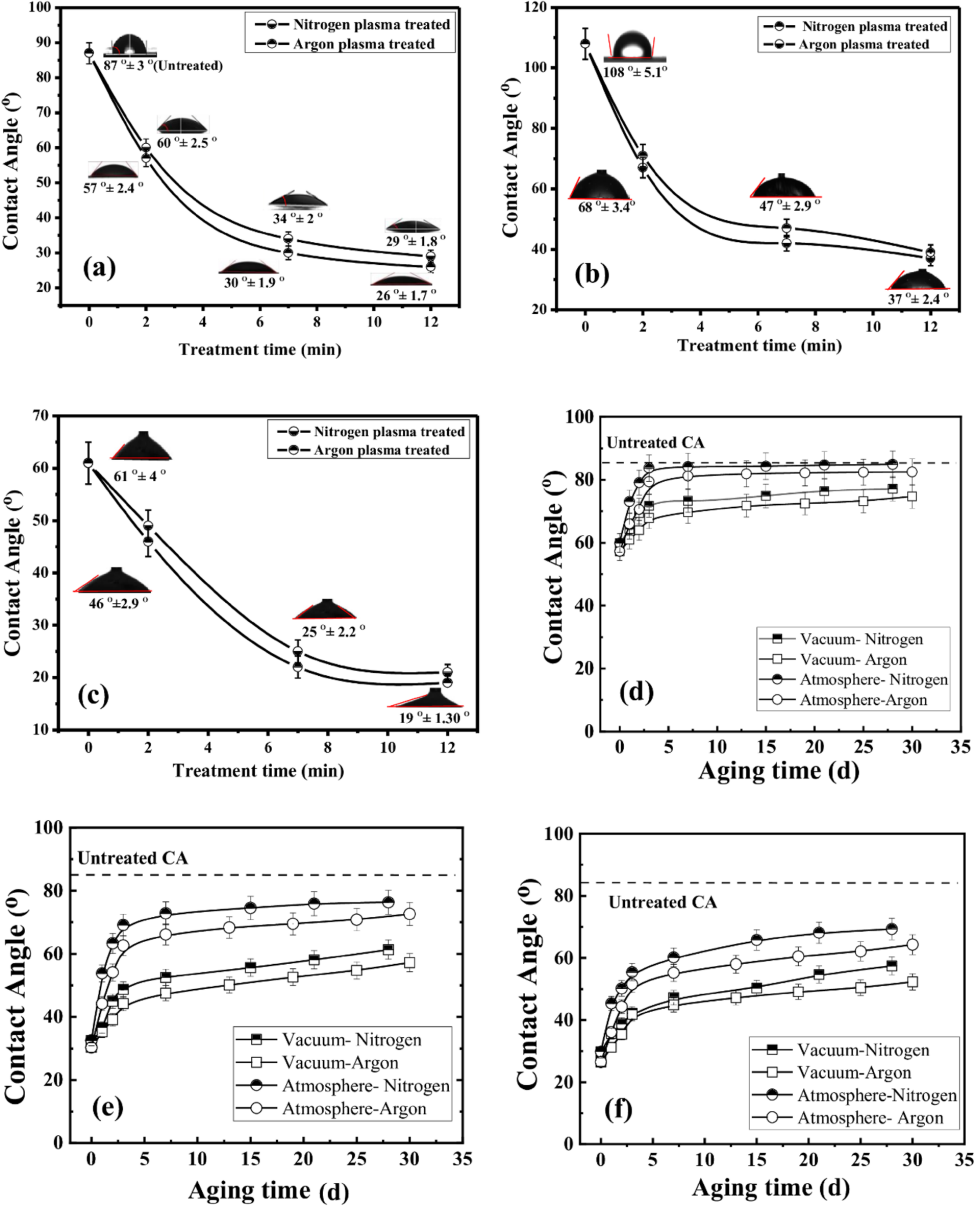
(**a**) Initial static contact angle variation after plasma treatment for different exposure time; (**b**) Initial advancing contact angle variation after plasma treatment for different exposure time, (**c**) Initial receding contact angle variation after plasma treatment for different exposure time; Variation of contact angle with ageing time for plasma treatment time: (**d**) 2 min, (**e**) 7 min, and (**f**) 12 min.

An A-Cam contact angle analyser is used to measure the water CA for both virgin and plasma-treated membranes for different plasma treatment times. The CA of the virgin membrane is found to be 87° ± 3° by using the sessile drop method. The CA, measured immediately just after the plasma treatment is considered as the initial CA of the plasma-treated membrane. Figure 1a–c illustrates the variation of initial static, advancing and receding CA with plasma treatment time for both N_2_ and Ar plasma-treated membrane. A strong dependence of CA on plasma treatment time is observed. It is found that the initial CA decreases prominently with an increase in plasma treatment time^43^.

To explore the influence of the storage medium on the ageing behaviour of plasma-treated membranes, the membranes are stored in two distinct environments—Atmosphere and Vacuum. In the atmospheric medium, membranes are kept in a desiccator at atmospheric pressure and room temperature throughout the study. Simultaneously, in the vacuum medium, membranes are stored within a cylindrical chamber made of SS-304L material (Diameter: 20 cm, Height: 20 cm). The vacuum pressure of the chamber is maintained at approximately 10^–2^ mbar using a rotary pump with a pumping speed of ~ 540 L/s. For a better understanding of the changes in CA of plasma-treated membranes over time, the CA of the stored membranes is measured at an interval of 1 h for the initial 72 h for both storage mediums. The initial CA and CA after 72 h for different plasma exposure times for atmospheric and vacuum mediums are shown in Table 1A and B. The initial static CAs for N_2_ plasma-treated membranes are found to be 60° ± 2.5°, 34° ± 2°, and 29° ± 1.8° for treatment times 2, 7 and 12 min, respectively. Similarly, for Ar plasma treatment, the initial CAs are found to be 57° ± 2.4°, 30° ± 1.9°, and 26° ± 1.9° for treatment times 2, 7, and 12 min, respectively. The decrease in CA with an increase in treatment time is attributed to surface etching and the introduction of hydrophilic components on the surface^1^. From Fig. 1a–c and Table 2, a slightly enhanced hydrophilic modification of membrane surface is observed for Ar plasma treatment in comparison to N_2_ plasma treatment. The higher nitrogen and oxygen functionalities in Ar and N_2_ plasma-treated membrane, as compared to untreated membrane, as observed from XPS analysis, are the main factors for enhancement of hydrophilicity for plasma-treated membrane. As the total oxygen and nitrogen components in the Ar plasma-treated membrane are found to be slightly higher as compared to the N_2_ plasma-treated membrane, the Ar plasma-treated membrane shows a better hydrophilic nature as compared to the N_2_ plasma-treated membrane. Additionally, the surface roughness also plays a vital role in water CA as well as surface hydrophilicity^1,24^. The increase in surface roughness in the Ar plasma-treated membrane, as compared to the N_2_ plasma-treated membrane, as observed from AFM analysis, is another factor in getting better hydrophilicity in Ar plasma treatment.

**Table 1:** Initial static CA and CA after 72 h for different storage mediums.

Sl. No	Storage medium	Initial CA			CA after 72 h		
		Static	Advancing	Receding	Static	Advancing	Receding
(A) For N_2_ plasma treatment							
1	Atmosphere						
	2 min	60° ± 2.5°	71° ± 3.7°	49° ± 3°	83.7° ± 3.5°	102° ± 5°	63° ± 3.6°
	7 min	34° ± 2°	47° ± 2.9°	25° ± 2.2°	72.8° ± 2.8°	86° ± 4.3°	51° ± 2.7°
	12 min	29° ± 1.8°	39° ± 2.5°	21° ± 1.5°	55.4° ± 2.6°	71° ± 3.2°	45° ± 2.3°
2	Vacuum						
	2 min	60° ± 2.5°	71° ± 3.7°	49° ± 3°	71.7° ± 2.9°	84° ± 4.5°	47° ± 3°
	7 min	34° ± 2°	47° ± 2.9°	25° ± 2.2°	48.7° ± 2.4°	68° ± 4°	35° ± 2.6°
	12 min	29° ± 1.8°	39° ± 2.5°	21° ± 1.5°	47.2° ± 2.2°	66° ± 3.6°	33° ± 2.4°
(B) For Ar plasma treatment							
1	Atmosphere						
	2 min	57° ± 2.4°	68° ± 3.4°	49° ± 3°	79.4° ± 3.1°	102° ± 5°	63° ± 3.6°
	7 min	30° ± 1.9°	42° ± 2.5°	25° ± 2.2°	62.6° ± 3°	86° ± 4.3°	51° ± 2.7°
	12 min	26° ± 1.7°	37° ± 2.4°	21° ± 1.5°	51.5° ± 2.3°	71° ± 3.2°	45° ± 2.3°
2	Vacuum						
	2 min	57° ± 2.4°	68° ± 3.4°	49° ± 3°	67.9° ± 2.7°	94° ± 4.8°	55° ± 3.2°
	7 min	30° ± 1.9°	42° ± 2.5°	25° ± 2.2°	44.2° ± 2.4°	67° ± 3.9°	28° ± 2.5°
	12 min	26° ± 1.7°	37° ± 2.4°	21° ± 1.5°	41.8° ± 2.1°	63° ± 3.5°	26° ± 2.4°

**Table 2:** Elemental analysis of untreated and plasma-treated membranes using XPS.

Plasma condition	Elemental composition (%)			Elemental ratio	
	O1s	N1s	C1s	O/C × 100%	N/C × 100%
Untreated	50.87	0.41	48.65	104.56	0.84
N_2_ plasma (12 min)	48.27	3.73	48	100.56	7.77
Ar plasma (12 min)	49.25	3.32	47.43	103.83	7

To understand the hydrophobic recovery, the CA of the plasma-treated membranes with different exposure times are examined for up to 30 days for two different storage mediums i.e., atmosphere and vacuum. After an initial 72 h of plasma treatment, the CA of the plasma-treated membrane is measured at intervals of 5 days up to a period of 30 days. Figure 1d–f represents the change in contact angle with ageing time for three different treatment times. The recent observations show that the hydrophobic recovery for both N_2_ and Ar plasma-treated membranes is very fast for the initial 7 days. Beyond 7 days of the ageing period, the rate of hydrophobic recovery becomes slower and tends to stabilize. For the atmospheric medium, the hydrophobic recovery is found to be faster compared to the vacuum medium.

In the context of the atmospheric medium, both temperature and relative humidity emerge as pivotal determinants influencing the hydrophobic recovery process, exhibiting temporal variations. The temperature-dependent dynamic stands out as a paramount factor governing the recovery of hydrophobicity in plasma-treated membranes^44–48^. The ageing effect of the plasma-treated membranes is very much sensitive to the storage temperature, and the fluctuations of storage temperature, mainly in the case of atmospheric medium, have instigated the chain motion during the post-plasma treatment stabilization of the membrane^44–48^. The increases in storage temperatures foster chain mobility, which in turn accelerates the ageing behaviour of the membrane^44–48^.

During post-plasma treatment, the system undergoes thermal activation as the temperature ascends, resulting in an augmented energy gain rate, proportional to ∼ *k*_*B*_T (where *k*_*B*_ denotes the Boltzmann constant, and T signifies the temperature in Kelvin). This temperature-dependent dynamic significantly impacts the migration and angular reorientation of low molecular weight chains and surface polar groups. Consequently, the polymeric membrane undergoes rapid hydrophobic recovery with an elevated temperature. Conversely, at lower temperatures, the rate of hydrophobic recovery diminishes owing to a reduction in the diffusion and reorientation dynamics^49^. The potential reactive groups for surface hydrophilization of polymeric surfaces are carboxyl, amine, amide, hydroxyl, etc.^50,51^. All groups contain oxygen. During the ageing process, these groups are reoriented and diffused to the bulk of the polymer. Hence, there is a reduction in the concentration of oxygen-containing polar functional groups on the surface, resulting in a decrease in the surface energy of the polymeric surface^52^. Thus, hydrophilicity introduced on the polymer surface starts decreasing, and CA increases. Due to such effects, the rapid hydrophobic recovery occurs in the case of atmospheric medium as compared to vacuum medium^53^.

Conversely, in the vacuum medium, the availability of potential reactants responsible for diffusion and reorientation reactions is significantly lower compared to the atmosphere. Consequently, the probability of reaction kinetics in a vacuum medium is notably reduced in comparison to an atmospheric medium. This lower probability results in a slowed-down recovery rate in the vacuum medium.

Recent observations indicate that in the atmospheric medium, the CAs after 28 days of plasma treatment are approximately 85° and 80° for N_2_ and Ar plasmas, respectively, for 2-min treatment time. For a 7-min treatment time, the CAs after 28 days of plasma treatment are approximately 76° and 72° for N_2_ and Ar plasmas, respectively. Again, for a 12-min treatment time, the contact angles after 28 days of plasma treatment are approximately 69° and 64° for N_2_ and Ar plasmas, respectively. The hydrophobic recovery of plasma-treated membranes for a 2-min treatment is significantly higher compared to 7-min and 12-min treatment time (refer to Fig. 1). As the change in hydrophilicity of the membrane for 7-min and 12-min is significant in comparison to treatment time 2-min, thus, the plasma-treated membrane with treatment time 7-min and 12-min are only utilized for further application studies.

### Ageing behaviour of the membrane using theoretical model prediction and experimental findings

In the plasma-treated polymeric membrane, the polar functional groups are introduced on the surface by plasma treatment and post-plasma functionalization. Hence, the hydrophilicity of the membrane surface increases as well as the water CA value decreases. As the storage time increases, the composition of the topmost surface at a bulk level experiences alteration. The newly introduced species on the membrane's topmost layer create a concentration gradient between the bulk and the top surface. As a result, a phenomenon known as the diffusion of different species takes place to compensate for the concentration gradient between the bulk and modified topmost layers. Hence, the membrane surface experiences a hydrophobic recovery process. During the process, the water CA value starts increasing as hydrophilicity decreases. Moreover, reorientation of the polar groups is also predicted within the bulk of the polymer, driven by translational and rotational motions, as suggested by previous studies^32,40,54–59^. This reorientation is a consequence of the modified membrane striving to achieve a state of minimum surface energy^32,40,54–59^. Thus, a rapid change in CA takes place until the of the post plasma stabilization process becomes insignificant.

**Figure 2:**
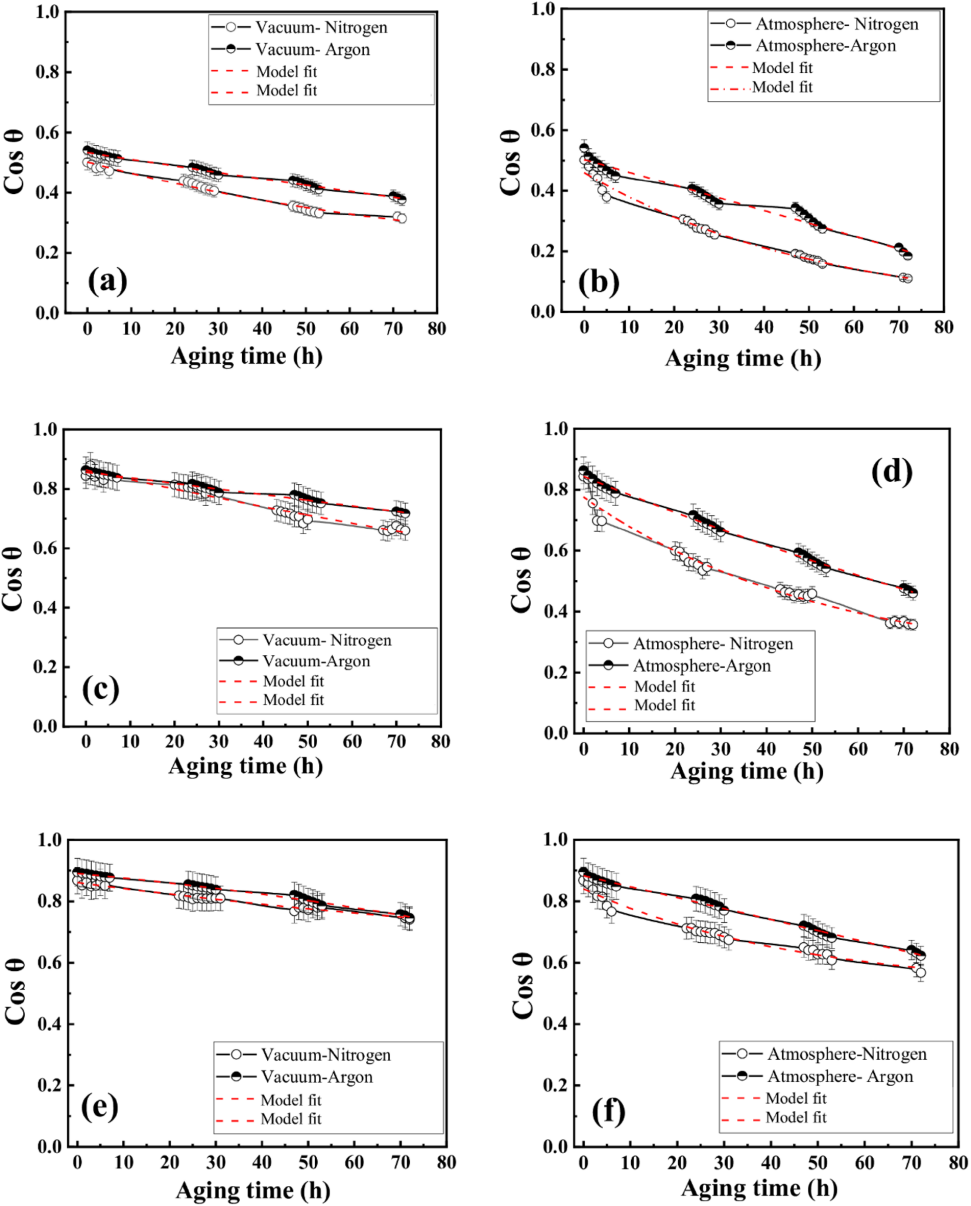
The model prediction and experimental data of the hydrophobic recovery; Storage in vacuum: (**a**) 2 min plasma exposure time, (**c**) 7 min plasma exposure time, (**e**) 12 min plasma exposure time; Storage in atmosphere: (**b**) 2 min plasma exposure time, (**d**) 7 min plasma exposure time, (**f**) 12 min plasma exposure time.

For a better understanding of the ageing behaviour, both N_2_ and Ar plasma-treated membranes are stored in atmospheric and vacuum mediums in the present work. Figure 2 represents the change in CA values in cosine function with the ageing period for both atmospheric and vacuum mediums. The diffusion model which is used to estimate the values of CA is discussed in the “Theoretical model for hydrophobic recovery” section. The experimental values of CA for both N_2_ and Ar plasma treatment are validated with the theoretical results and presented in Fig. 2. It is clearly observed that the experimental results show a very good agreement with the theoretical results.

### Surface morphology

The surface modification of the membrane due to plasma etching^4,24^ plays a crucial role in removing lightweight molecular species like additives, processing aids, and adsorbed materials from the surface. The effectiveness of this phenomenon is highly dependent on the choice of operating gas used for plasma generation. In addition to the chemical reactivity of plasma species, the inherent nature of the polymer significantly influences the etching rate.

**Figure 3:**
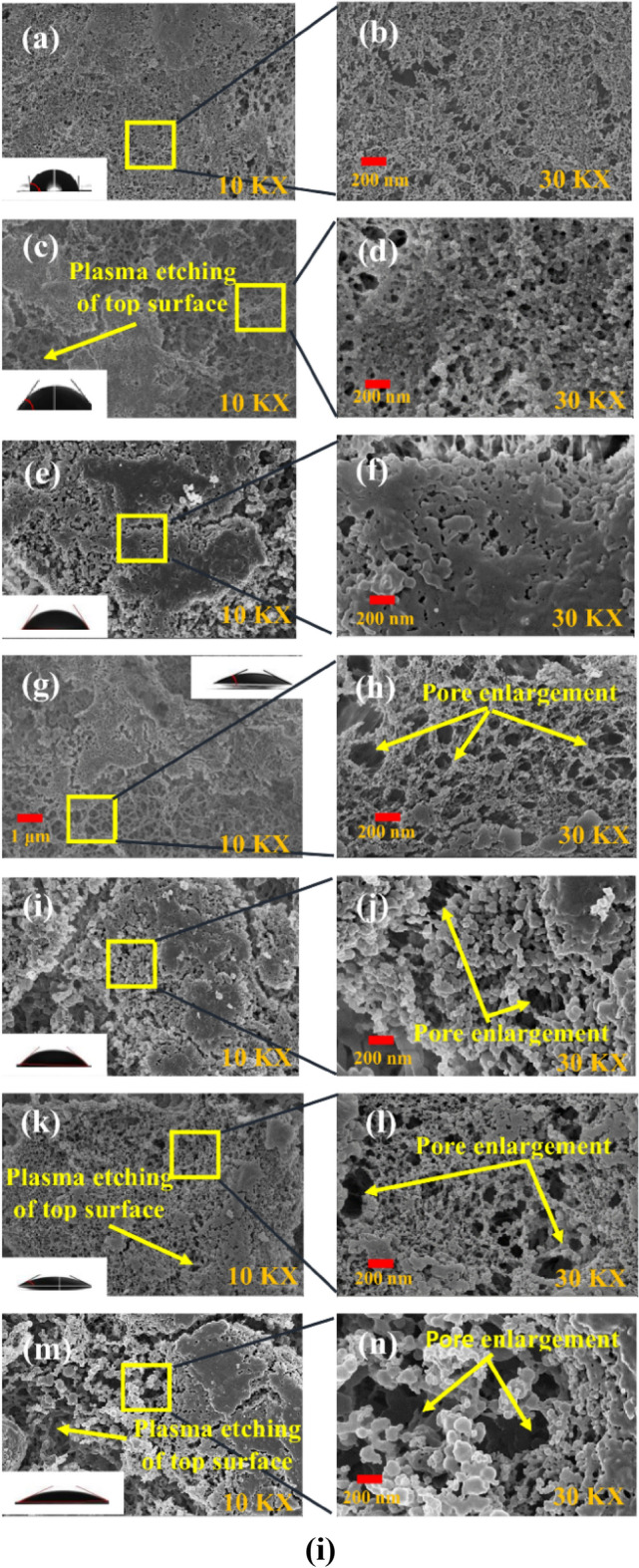

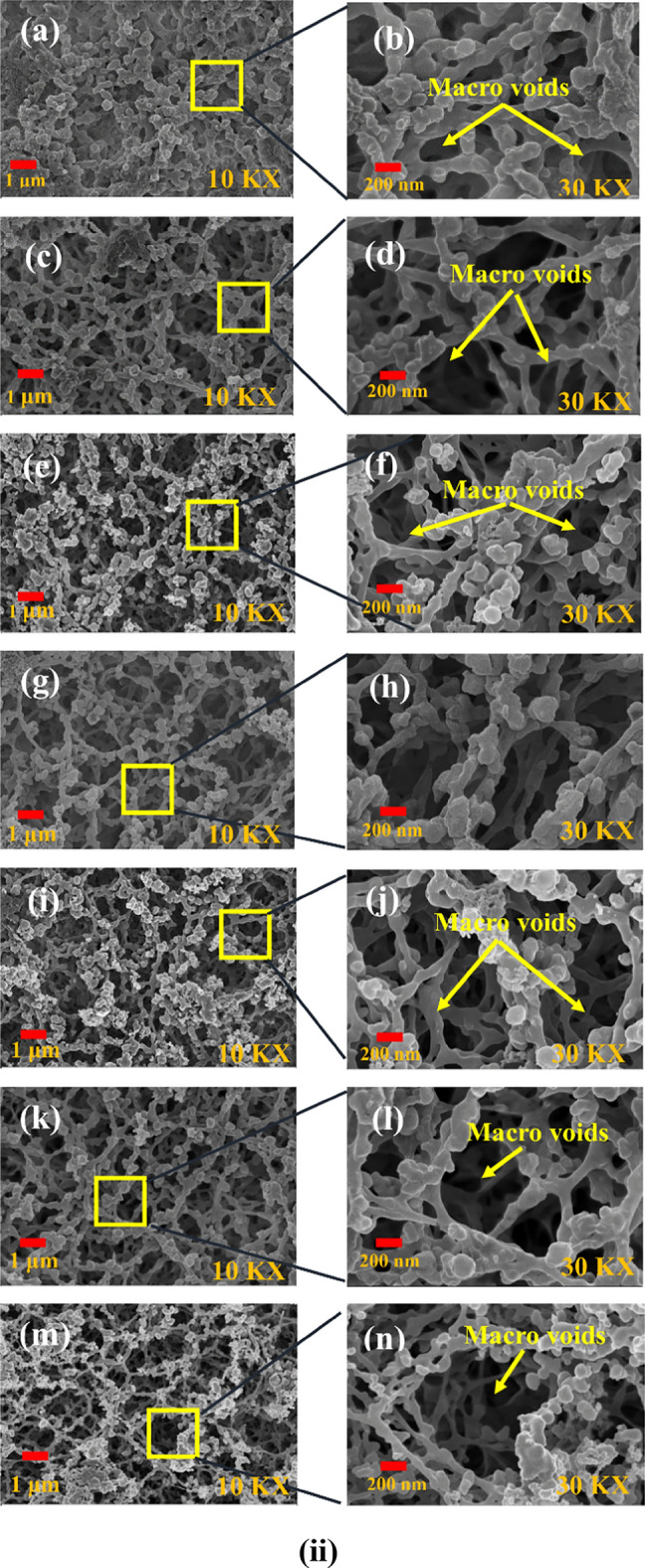
SEM pictures of membranes (**i**) top surface: (**a**, **b**) untreated membrane [Reproduced from Ref. 24 with permission, Copyright 2023 John Wiley & Sons]; (**c**, **d**) and (**e**, **f**) are the N_2_ and Ar plasma-treated (2 min exposure time); (**g**, **h**) and (**i**, **j**) are the N_2_ and Ar plasma-treated (7 min exposure time) and (**k**, **l**) and (**m**, **n**) are the N_2_ and Ar plasma treated (12 min exposure time); (**ii**) bottom surface: (**a**, **b**) untreated membrane [Reproduced from Ref. 24 with permission, Copyright 2023 John Wiley & Sons]; (**c**, **d**) and (**e**, **f**) are the N_2_ and Ar plasma-treated (2 min exposure time); (**g**, **h**) and (**i**, **j**) are the N_2_ and Ar plasma-treated (7 min exposure time) and (**k**, **l**) and (**m**, **n**) are the N_2_ and Ar plasma-treated (12 min exposure time).

Figure 3i and ii show the SEM images of the top and bottom surfaces of the virgin and plasma-treated membranes with Nitrogen and Argon plasmas. Here, the SEM images of the untreated and plasma-treated membrane are presented for two magnifications, i.e., for 10 kX and 30 kX. In the present work, the SEM characterization is carried out after 20 days of ageing for atmospheric storage conditions^4^.

The top view of the virgin membrane is shown in Fig. 3(i)-(a,b). The Nitrogen and Argon plasma-treated polymeric membranes for different exposure times are shown in Fig. 3(i)-(c–n). The surface roughness, along with the pore diameters, is evidently increased for both Nitrogen and Argon plasma treatments at different exposure times compared to the untreated membrane. A very prominent pore enlargement and plasma etching effect have been observed in Fig. 3(i)-(g,h) for N_2_ plasma-treated membrane and Fig. 3(i)-(i,j) for Ar plasma-treated membrane for an exposure time of 7 min. For an exposure time of 12 min, the SEM images are shown in Fig. 3(i)-(k,l) for the N_2_ plasma-treated membrane and Fig. 3(i)-(m,n) for the Ar plasma-treated membrane, respectively. From the SEM images, it is clearly visible that the pore enlargement and plasma etching effect for the top surface of the plasma-treated membranes are very significant for 7 min and 12 min of exposure time compared to 2 min, as shown in Fig. 3(i)-(c,d) and (e,f).

The bottom of the virgin membranes is depicted in Fig. 3(ii)-(a,b). In contrast, Fig. 3(ii)-(c–n) showcase the N_2_ and Ar plasma-treated membranes for different plasma treatment times. Notably, the formation and enlargement of macro voids are clearly evident in Fig. 3(ii) for both the plasma-treated membrane. A distinct increase in the size of macro voids is observed for plasma treatment durations of 7 min and 12 min compared to 2 min plasma treatment for both N_2_ and Ar plasma treatments.

The current study establishes that the plasma-exposed side i.e., the topmost surface of the membrane, undergoes effective modification during the treatment process. The hydrophilicity of the membranes increases proportionally with the enlargement of pore and void sizes. The experimental findings strongly suggest that the plasma treatment time significantly influences hydrophilicity of the treated membrane due to prominent surface modification as a result of plasma etching.

**Figure 4:**
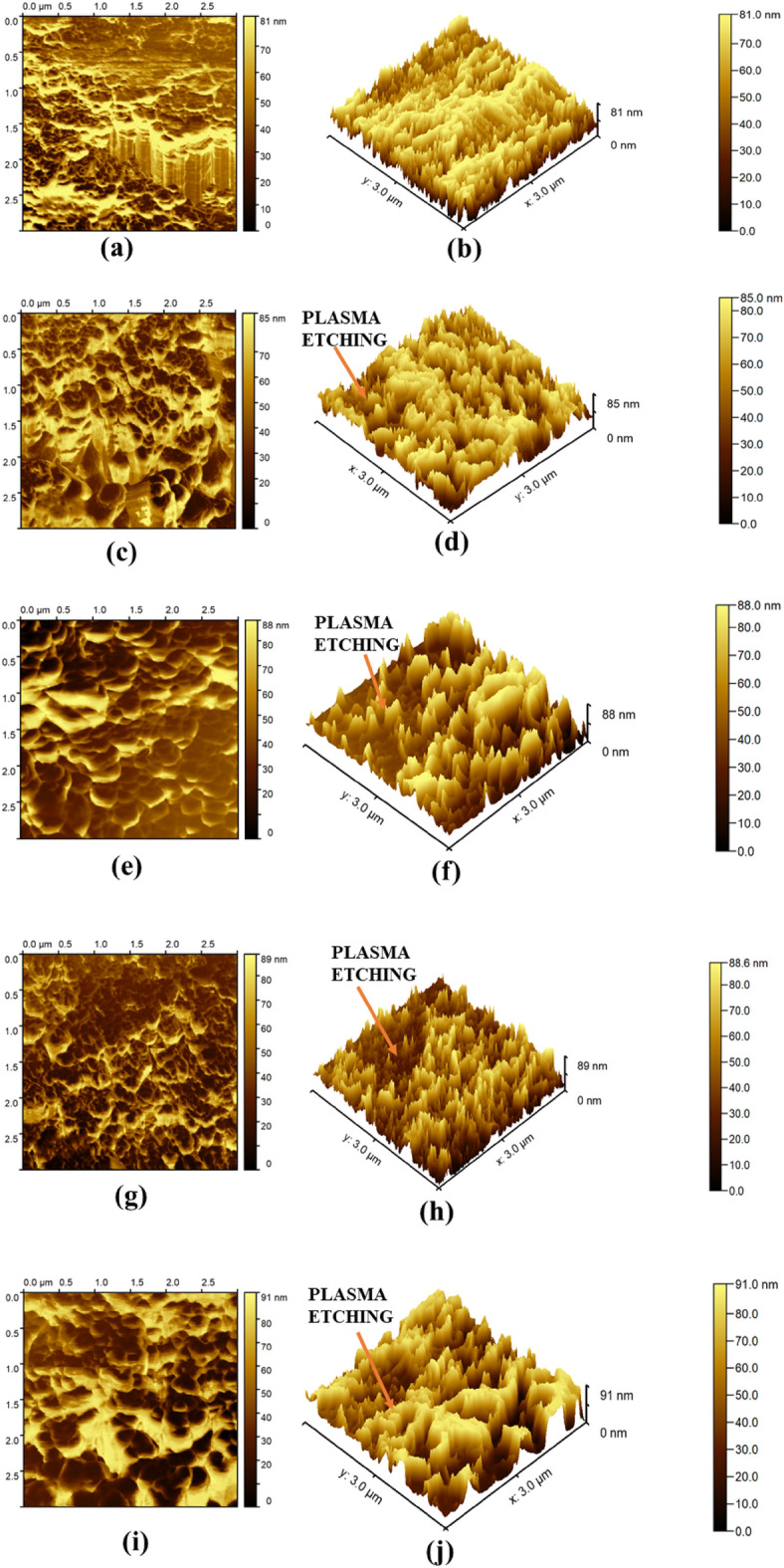
AFM 2D and 3D images of the membranes: (**a**, **b**) for untreated membrane; (**c**, **d**) for N_2_ plasma-treated membranes and (**e**, **f**) for Ar plasma-treated membranes (7 min exposure time); (**g**, **h**) for N_2_ plasma-treated membranes, and (**i**, **j**) for Ar plasma-treated membranes (12 min exposure time).

Due to plasma etching, the surface roughness of the plasma-treated membrane undergoes alteration^1,32,54–56^. In the present study, AFM analysis is conducted after 20 days of ageing for atmospheric storage conditions to investigate the surface roughness of both treated and untreated membranes. Figure 4a and b displays the AFM images of the virgin polymeric membrane. Conversely, Fig. 4c–f show the AFM images of N_2_ plasma and Ar plasma-treated membranes for a treatment time 7 min. Similarly, Fig. 4g–j represent the AFM images of N_2_ plasma and Ar plasma-treated membranes for a treatment time of 12 min, respectively.

Based on the analysis, the Root Mean Square (RMS) roughness for the untreated membrane is determined to be 19 nm. It is observed that due to N_2_ and Ar plasma treatment, the RMS roughness of the treated membrane increases substantially. For a treatment time of 7 min, the RMS roughness is measured as 27 nm and 28.5 nm for N_2_ and Ar plasma, respectively. Moreover, for a treatment time of 12 min, the RMS roughness further increases to 29 nm and 31 nm for N_2_ and Ar plasma, respectively. This increase in RMS roughness is attributed to the plasma etching and pore size enlargement, which is clearly observed in FESEM images^54,57^.

Comparing the Ar plasma and N_2_ plasma treatment, it is found from the SEM and AFM analysis that the Ar plasma treatment shows higher pore size enlargement and surface roughness in comparison to the N_2_ plasma treatment. This disparity may be attributed to various factors, including atomic mass, ionization degree, etching rate, etc. of the plasma process gas. Ar, with its higher atomic mass, ionization degree, and etching capability compared to N_2_, possesses distinct advantages in increasing the pore size and surface roughness of the membrane. These properties make Ar plasma more effective in modifying the surface of polymeric membranes, resulting in the observed improved outcomes in surface morphology and roughness.

### Surface chemistry

**Figure 5:**
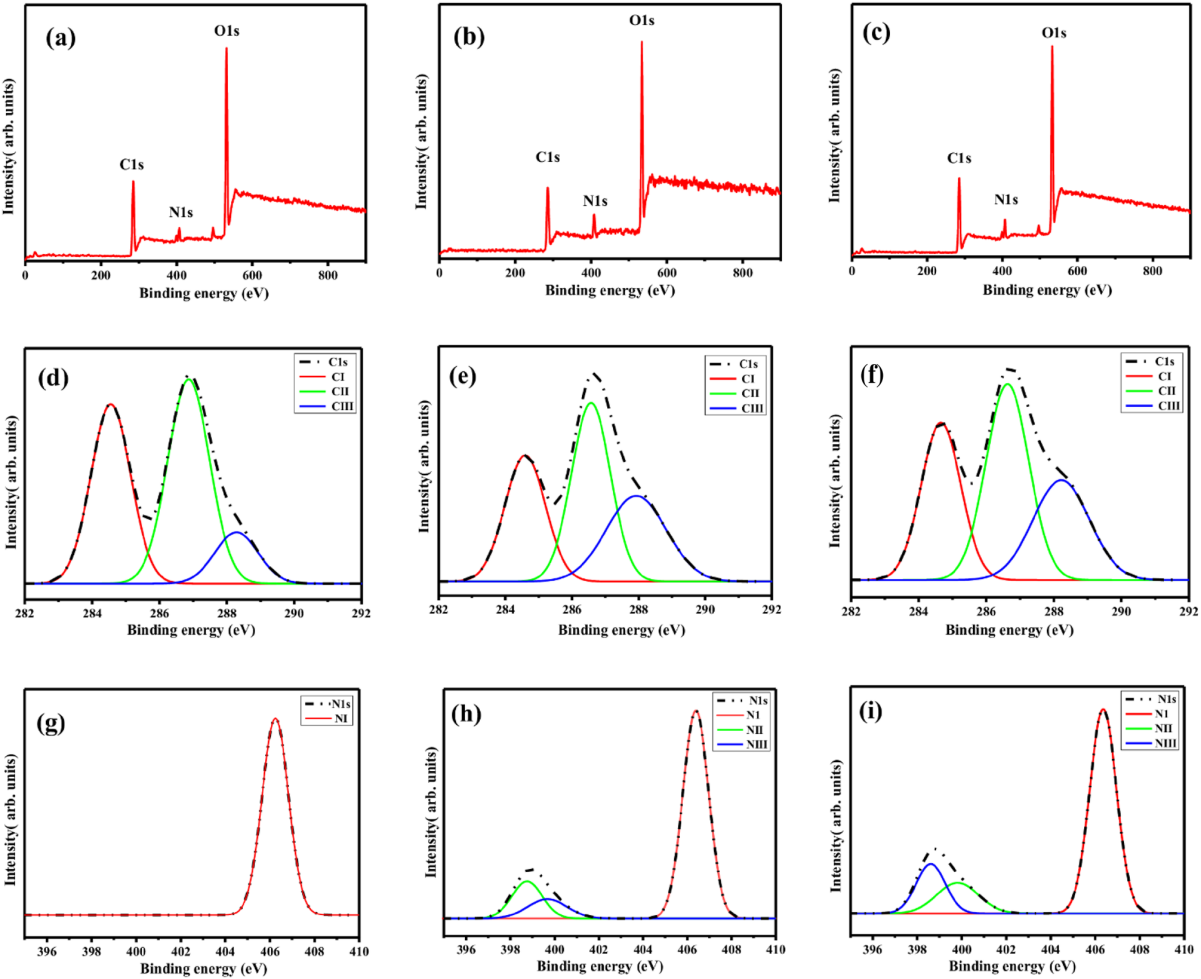
XPS survey spectra: (**a**) Untreated, (**b**) N_2_ plasma-treated, and (**c**) Ar plasma-treated membranes. Elemental spectra of C1s: (**d**) Untreated, (**e**) N_2_ plasma-treated, and (**f**) Ar plasma-treated membranes; Elemental spectra of N1s: (**g**) Untreated, (**h**) N_2_ plasma-treated, and (**i**) Ar plasma-treated membranes.

In this investigation, X-ray photoelectron spectroscopy (XPS) analysis assesses the chemical compositions of both the pristine and plasma-treated membrane surfaces. To ensure the stability of the elemental composition, XPS scans are conducted on the plasma-treated membranes after a 20-days of ageing process. The XPS spectra corresponding to untreated membranes and those treated with both N_2_ and Ar plasmas are depicted in Fig. 5a–c. The elemental compositions, as determined through survey scans conducted before and after plasma treatment, are succinctly presented in Table 2.

From Table 2 and Fig. 5a–c, it is found that a slight decrease in the intensity of C1s and O1s peaks for the membrane surface after N_2_ plasma treatment, in comparison to the untreated membrane. However, a significant increase in the N1s peak is observed for the N_2_ plasma-treated membrane. It indicates the incorporation of nitrogen-containing species into the polymer matrix during and post-plasma treatment^4^.

A modest change in the intensity of C1s and O1s peaks compared to the untreated membrane is also observed for Ar plasma treatment. Compared to the untreated membrane, a significant increase in the N1s peak is also observed for the Ar plasma-treated membrane. In the case of Ar plasma, the increase in N1s intensity might be due to the post-plasma functionalization effect, where the membrane, subjected to plasma treatment, undergoes atmospheric conditions characterized by a high probability of contact with nitrogen-containing molecules. This phenomenon is attributed to the fact that Ar plasma can activate the membrane surface^58^.

In comparison to the N_2_ plasma-treated membrane, the concentration of C1s and N1s are slightly decreased for Ar plasma-treated membrane, whereas the concentration of O1s is slightly increased for Ar plasma-treated membrane.

The concentration analysis of chemical components involving C1s and N1s is conducted through deconvolution using a Gaussian–Lorentzian fit. Maintaining almost consistent full-width at half maximum for all synthetic peaks, the observed functional groups after deconvolutions, along with their weight percentages, are summarized in Table 3. Figure 5d–f illustrates the deconvoluted peaks of C1s for the untreated membrane, N_2_ plasma-treated membrane, and Ar plasma-treated membrane, respectively. For the C1s peak, three distinct deconvoluted peaks are observed at 284.7 ± 0.2 eV (CI), 286.6 ± 0.3 eV (CII), and 288.6 ± 0.3 eV (CIII) for both the untreated and plasma-treated membranes. The peaks at 284.7 ± 0.2 eV correspondence for C–C or C–H group, 286.6 ± 0.3 eV to either C–O or C–N group, and 288.6 ± 0.2 eV to O–C=O group^1,4,26,59–62^. For CII peaks, multiple functional groups are possible and individual detection is challenging.

**Table 3:** XPS data for untreated and plasma treated membrane.

Group assignment	Binding energy (eV)	% Concentration		
		Untreated	N_2_ plasma treated	Ar plasma treated
C1s				
C–C/C–H (CI)	284.7 ± 0.2	39.9	29.7	30.8
C–O/C–N (CII)	286.6 ± 0.3	47.8	40.5	41.4
O–C=O (CIII)	288.6 ± 0.3	12.3	29.8	27.8
N1s				
–NH–/C–N–C (NIII)	398.8 ± 0.2	0	17.5	10.5
C–N=O (NII)	400.3 ± 0.2	0	16	15.5
–NO_2_ (NI)	406 ± 0.3	100	66.5	74

Table 3 indicates that C–C or C–H group is decreasing by 26% and 23% after N_2_ and Ar plasma treatment, respectively. On the other hand, C–O or C–N group is decreasing by 15% and 13% after N_2_ and Ar plasma treatment, respectively. Lastly, it is found an increment of 2.4 and 2.2 times for O–C=O group N_2_ and Ar plasma-treated membrane, respectively.

Figure 5g–i represents the deconvoluted peaks of the N1s peak for untreated, N_2_ plasma-treated and Ar plasma-treated membranes, respectively. Initially, the untreated N1s peaks contain only one significant peak at 406 ± 0.3 eV corresponding to either ${\text{NO}}_{2}$ (NI) group^63^. However, post plasma treatment two additional peaks emerge at 398.6 ± 0.2 eV and 400.2 ± 0.1 eV, respectively indicating the either NH or C–N–C (NIII) and C–N=O (NII) groups^4,62–66^. Following the N_2_ plasma treatment, the ${\text{NO}}_{2}$ groups decrease by 33.5%. In contrast, either NH or C–N–C (NIII) and C–N-O groups increase by 17.5% and 16.5%, respectively. Contrastingly, with Ar plasma treatment, the ${\text{NO}}_{2}$ groups experience a reduction of 26%. Conversely, either NH or C–N–C (NIII) and C–N=O groups show an increase of 10.5% and 15.5%, respectively.

**Figure 6:**
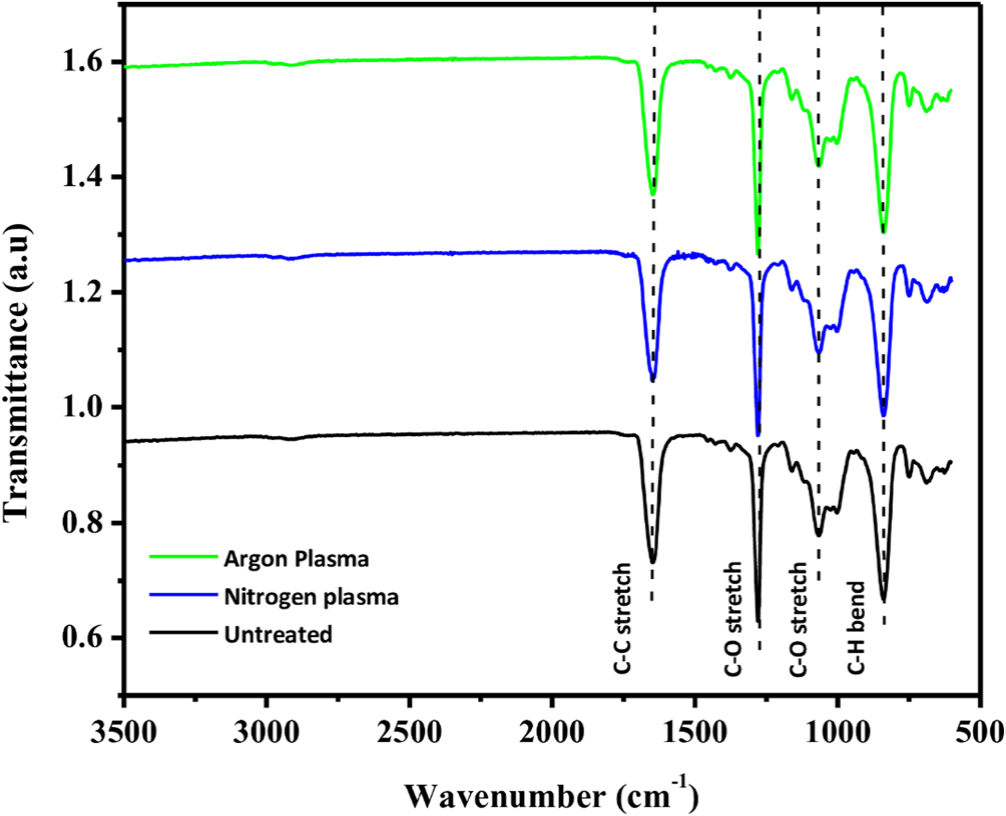
FTIR spectra of virgin and Plasma-treated (N_2_ and Ar plasma, 12 min treatment time) membrane.

Additionally, in the present work Fourier Transform Infrared (FTIR) spectroscopy is employed to conduct a comprehensive analysis of the structural alterations in both virgin and plasma-modified membranes for both Ar plasma and N_2_ plasma. Figure 6 displays the recorded spectra of the virgin and plasma-treated polymeric membranes. Several distinct peaks are observed in both the virgin and plasma-treated membranes. Specifically, the peaks appearing at 1643 cm^−1^ correspond to C–C stretch, while those at 1275 cm^−1^ and 1059 cm^−1^ correspond to C-O stretch, and the peak at 834 cm^−1^ corresponds to C–H bend.

Interestingly, the FTIR spectra exhibit similar peaks for both the N_2_ and Ar treated membranes, resembling the untreated membrane. This similarity indicates that the bulk properties of both the N_2_ and Ar treated membranes remain largely unchanged compared to the untreated membrane. The plasma treatment has a significant impact on the surface morphology and surface chemistry of the topmost surface of the membrane while leaving the bulk properties relatively unaffected^24^. This is attributed to the fact that the depth of modification provided by the plasma treatment is lower than the penetration depth of the FTIR, thus resulting in similar FTIR spectra for both the N_2_ and Ar treated membranes, resembling the untreated membrane^67^.

### Pore characterization of the untreated and plasma-treated membrane

**Figure 7:**
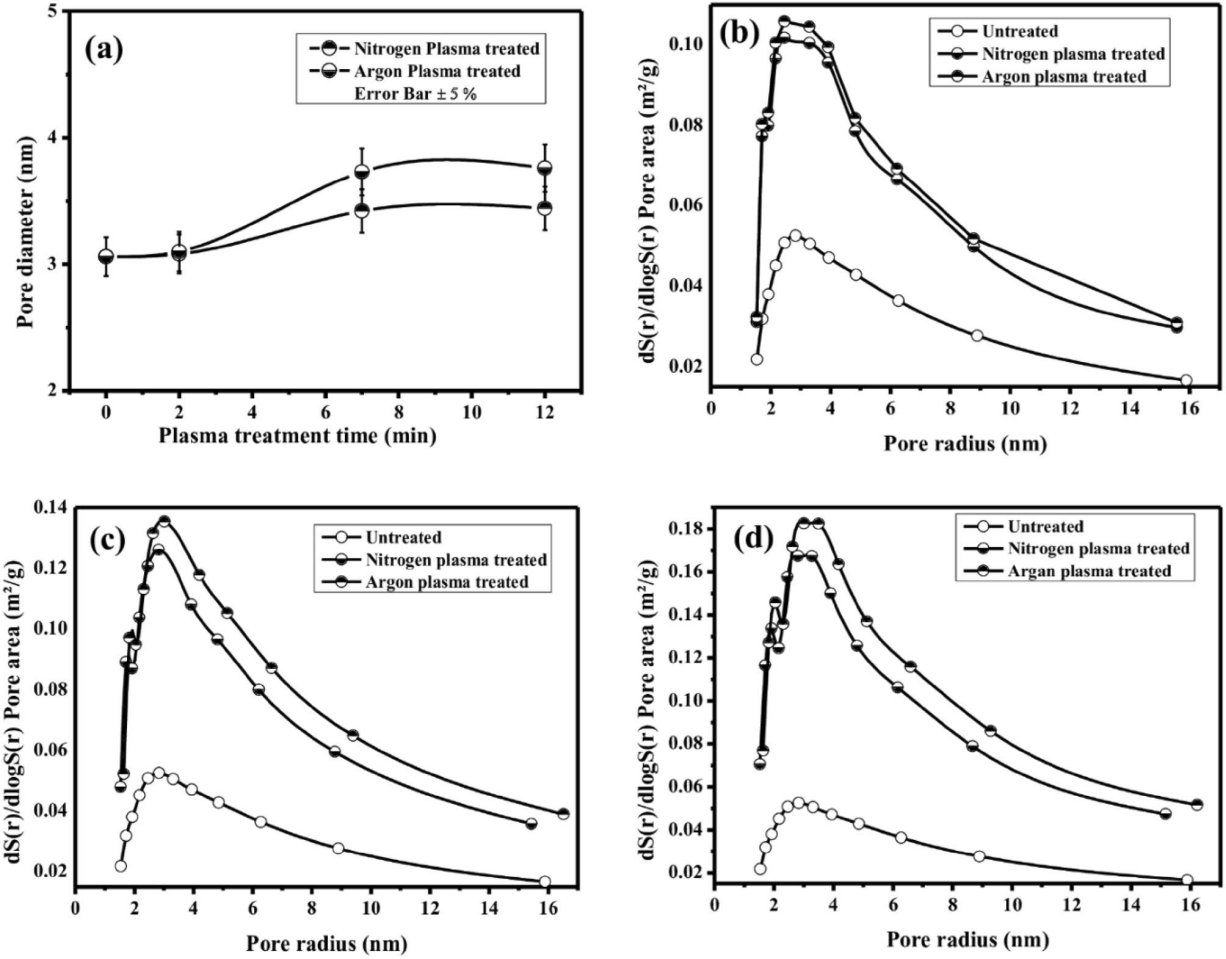
(**a**) Variation of pore diameter with plasma treatment time of virgin and Plasma-treated (N_2_ and Ar gas) membranes; Pore area distribution and comparison of virgin and Plasma-treated (N_2_ and Ar gas) membranes: (**b**) For 2 min treatment time, (**c**) For 7 min treatment time, and (**d**) For 12 min treatment time.

The pore size distribution for untreated and plasma-treated membranes is carried out using the Barrett-Joyner-Halenda (BJH) method at 77 K after 20 days of ageing for atmospheric storage conditions. The pore size distribution of untreated membranes and those treated with N_2_ and Ar plasma (at different treatment durations of 2, 7 and 12 min) is illustrated in Fig. 7a. Figure 7a indicates that the pore diameter of the untreated membrane is 3 nm. In contrast, for N_2_ plasma treatment (For treatment time: 12 min), the pore diameter increases to 3.44 nm, and for Ar plasma treatment (For treatment time: 12 min), the pore diameter further increases to 3.76 nm. Thus, an approximately 14.7% increase in pore size is observed for N_2_ plasma treatment, whereas an approximately 25.34% increase in pore size is observed for Ar plasma treatment. Remarkably, there is a significant enhancement of 10.64% in the pore diameter of the membrane for Ar plasma treatment compared to N_2_ plasma treatment.

In Fig. 7b–d; the pore distribution variation for both the N_2_ and Ar plasma treatments is presented with respect to plasma treatment times of 2, 7 and 12 min, respectively. The figures demonstrate that as the plasma treatment time increases, both the average pore area and the average pore diameter are increased. Additionally, a notable increment in pore area and pore radius is observed for Ar plasma treatment compared to N_2_ plasma treatment. This phenomenon can likely be attributed to the higher etching rate during Ar plasma treatment, resulting from the higher atomic weight and ionization degree of Argon compared to Nitrogen.

### Separation, antifouling performance and rejection rate of untreated and plasma-treated membranes

In the present work, a gravity filtration setup at atmospheric pressure and room temperature is used to determine the permeate flux. Both the N_2_ and Ar plasma-treated membranes (For 7 min and 12 min treatment time) along with the untreated membranes are used to perform the antifouling test for mud water. The Mud water consists of 20 g/L of sludge^68^.

**Figure 8:**
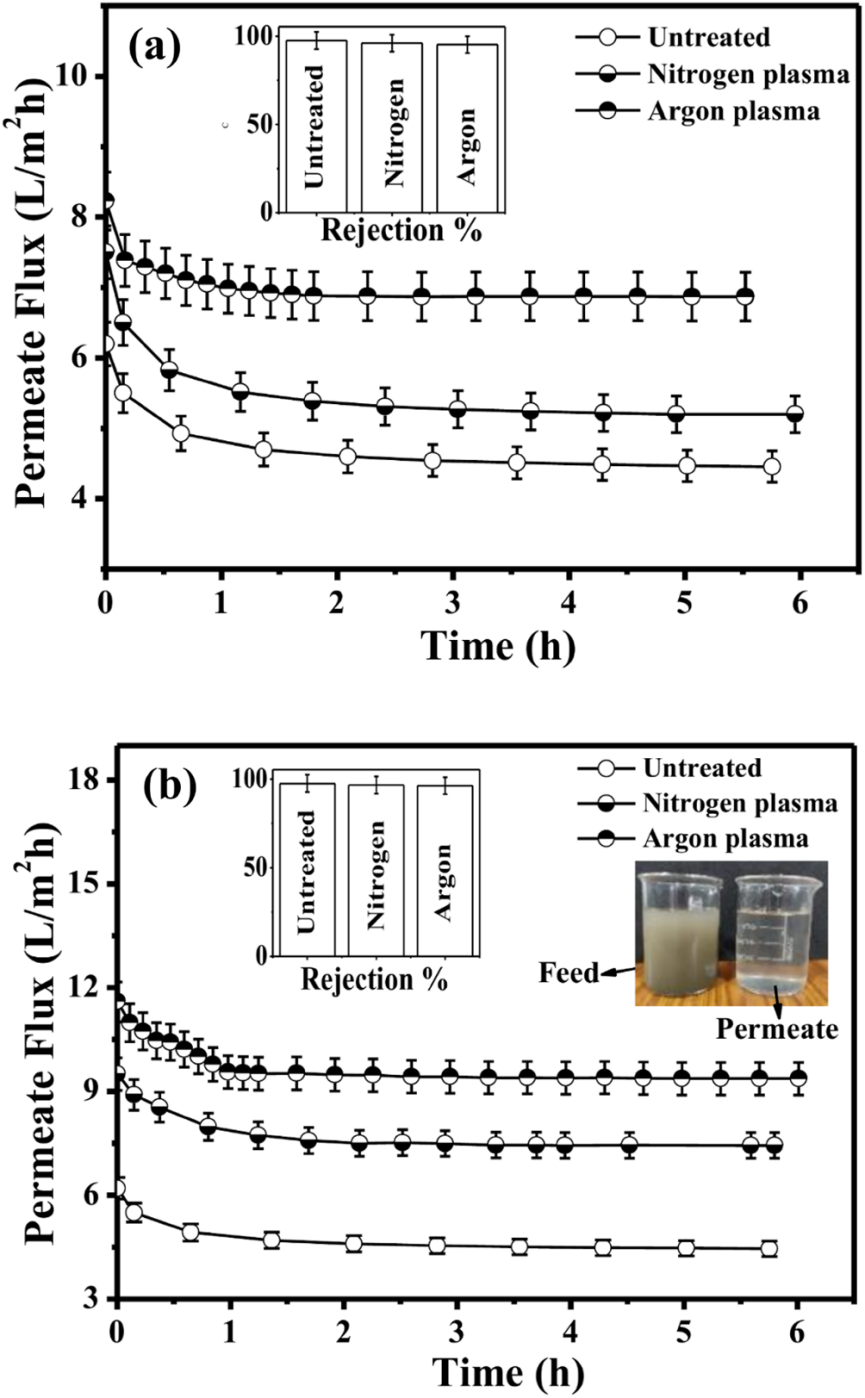
Variation of permeate flux with rejection rate for untreated and plasma-treated membranes: (**a**) 7 min exposure time and (**b**) 12 min exposure time for mud water with concentration of 20 g/L of sludge.

The impact of plasma treatment on the membrane flux over a filtration duration of 6 h is discernible through Fig. 8a and b. A significant improvement in permeation flux of CN membranes is observed for both N_2_ and Ar plasma-treated membranes for a treatment time of 7 min and 12 min in comparison to the untreated membrane. It can be observed from Fig. 8 that the permeate flux of the membrane is increased by 2.63 times after Ar plasma treatment for a treatment time of 12 min, whereas in the case of N_2_ plasma treatment for a treatment time of 12 min, the enhancement of flux is 1.21 times in comparison to the untreated membrane. For 7 min of treatment time, the permeate flux is increased by 1.54 times for the Ar plasma-treated CN membrane in comparison to the untreated membrane. For a similar treatment time i.e. 7 min, the enhancement of permeate flux for N_2_ plasma-treated CN membrane is 1.17 times in comparison to the untreated membrane. The longer plasma treatment time leads to the introduction of more hydrophilic functionality on the membrane surface, resulting in a significant improvement in permeation flux.

Additionally, Ar plasma treatment shows better permeate flux for mud water in comparison to N_2_ plasma treatment. The increase in permeate flux of mud water for Ar plasma treatment in comparison to N_2_ plasma treatment might be attributed to two main factors: (i) a rise in pore size and/or (ii) an augmentation in membrane hydrophilicity after plasma treatment. The improved permeability can be attributed to the heightened permeation caused by enhanced hydrophilicity. Consequently, the enhancement in permeate flux during post-plasma treatment validates the hydrophilization of the treated membrane.

### Antifouling model of untreated and plasma-treated membranes

**Figure 9:**
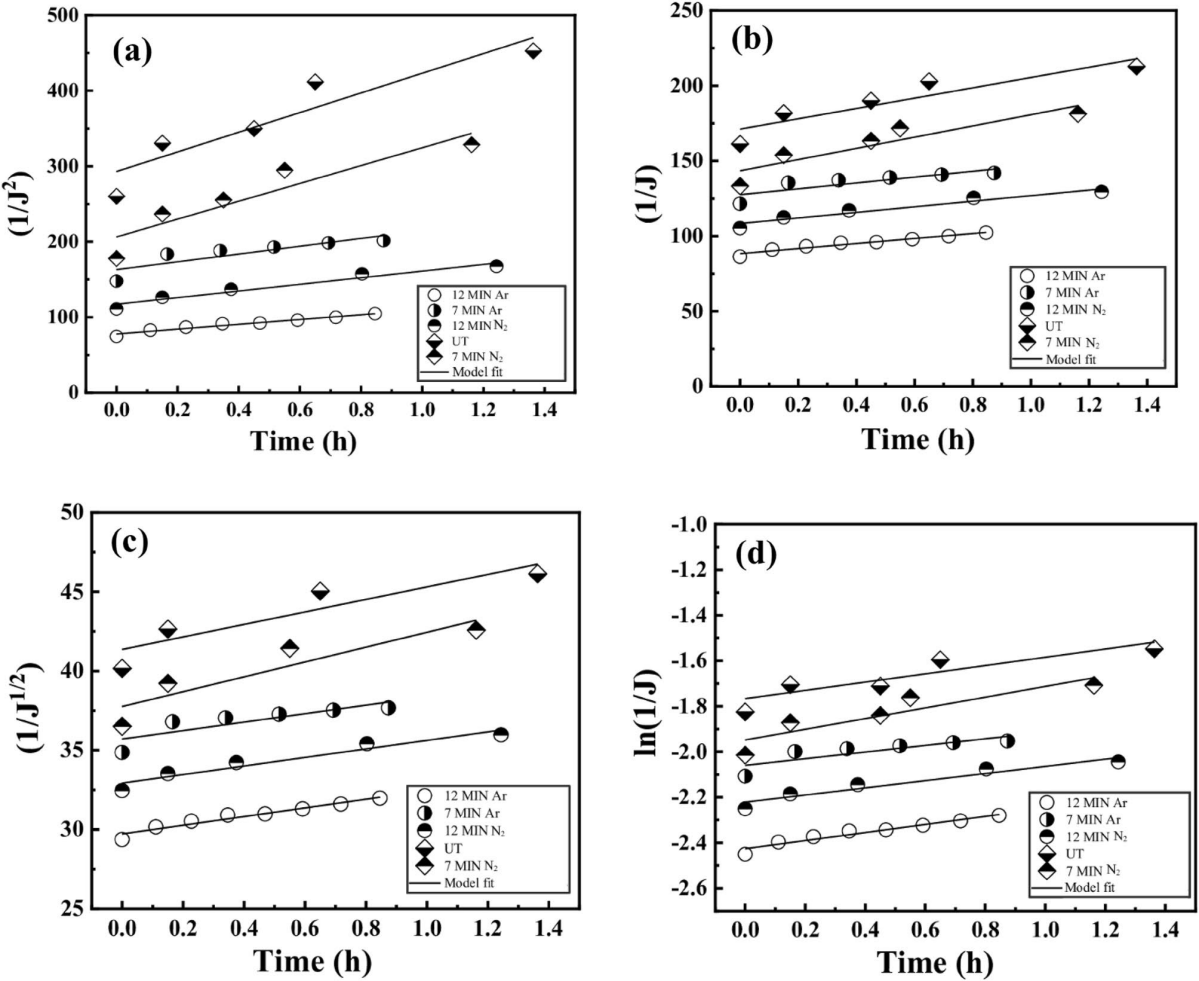
The characteristic of the fitting equation for dead-end filtration: (**a**) complete pore blocking model, (**b**) intermediate pore blocking model, (**c**) standard pore-blocking model, and (**d**) cake filtration model.

To determine the most suitable Hermia model for describing fouling mechanisms, the key criterion is to identify the model with the highest R^2^ value among the untreated and the plasma-treated membranes. The Cake Filtration Model (n = 0) consistently emerges as the most fitting model for determining fouling mechanisms across untreated and plasma-treated membrane filtration, as evidenced by the highest coefficient of fitting of Hermia’s Model i.e., R^2^ values as shown in Table 4 and Fig. 9.

**Table 4:** Coefficient of fitting of Hermia’s model.

Model of antifouling mechanism	Untreated CN	N_2_ Plasma treated CN (12 min)	N_2_ Plasma treated CN (7 min)	Ar Plasma Treated CN (12 min)	Ar Plasma Treated CN (7 min)
Cake filtration model (n = 0)	0.88	0.95218	0.86922	0.9732	0.74697
Intermediate *pore-blocking* model (n = 1)	0.85	0.93977	0.84128	0.96591	0.72727
Standard *pore-blocking model* (n = 1.5)	0.83	0.93295	0.82649	0.96144	0.71742
Complete *pore-blocking* model (n = 2)	0.82	0.92573	0.81122	0.95644	0.70758

This model implies that the primary fouling mechanism involves the accumulation of a filter cake on the membrane surface, where the rate of flux decline is directly associated with cake thickness. The robust fit of this model underscores the significance of cake formation as the predominant fouling process in these scenarios. The initial formation of a densely concentrated fouled cake layer on the membrane surface triggers a sharp decline in flux, effectively acting as a secondary filtration barrier^69^. During the initial filtration stages, both small and large particles present in the feed water adhere to the membrane surface, resulting in a pronounced decrease in permeate flux. As time progresses, the rate of particle deposition slows, leading to a stabilization in filtration rate^70^. Concurrently, the cake layer operates as an additional filtration stratum on the membrane surface. The size of the solute significantly influences the fouling cake's formation. Most solute sizes exceeded the membrane's average pore size, making cake filtration the primary fouling mechanism in this study.

The probable mechanisms driving cake filtration are^71^; cake formation: accumulation of particles on the filter medium forms a layer known as the filter cake; particle capture: particles in the filtered fluid are captured on the filter medium; pore blocking: particles block the pores in the filter medium, reducing the flow rate; cake compression: the cake undergoes compression, diminishing its permeability and cake consolidation: over time, the cake may undergo consolidation or compaction, altering its filtration properties.

### Plasma characterization

Langmuir probe^72,73^ and Optical Emission Spectroscopy (OES) are used to characterize the plasma in the recent work. A cylindrical Langmuir probe (Length: 10 mm and Dia: 0.25 mm) is used in the recent experiment to characterize both Ar plasma and N_2_ plasma in the presence and absence of the membrane. It is found that the plasma density (N_e_) for Ar plasma in the absence of the membrane is 4.65 × 10^15^/m^3^, and the electron temperature (T_e_) is 1.343 eV. However, in the presence of the membrane, the Ar plasma density is slightly decreased to 4.28 × 10^15^/m^3^ and no significant change is observed for electron temperature (T_e_ = 1.348 eV). For N_2_ plasma, the plasma density (N_e_) is found to be 3.42 × 10^15^/m^3^, and electron temperature (T_e_) is 1.481 eV in the absence of the membrane. In the presence of the membrane, the N_2_ plasma density is slightly decreased to 3.18 × 10^15^/m^3^, and electron temperature remains almost unchanged (T_e_ = 1.488 eV). The decrease in plasma density in the presence of the membrane might be due to the loss of plasma on the membrane.

**Figure 10:**
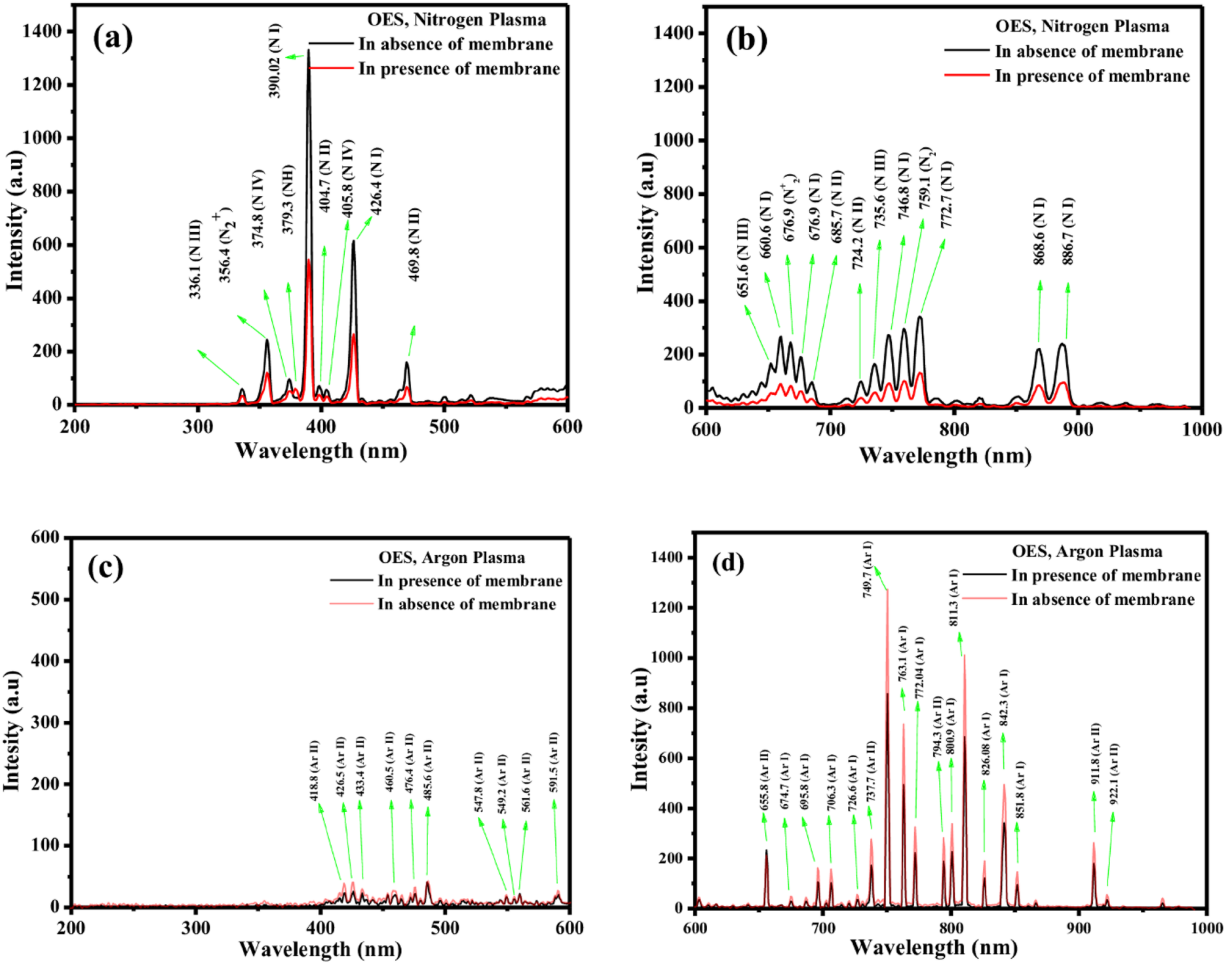
OES spectral profile of N_2_ Plasma (**a**, **b**) and Ar Plasma (**c**, **d**).

Optical Emission Spectroscopy (OES) stands as a widely employed analytical technique to monitor the spectral line intensities of the plasma^74^. In this current study, OES is used to record the spectral line profiles of DC glow discharge N_2_ and Ar plasma. The OES spectra for both the N_2_ and Ar plasma are meticulously presented in Fig. 10. Figure 10a and b portrays the spectra of N_2_ plasma in the presence and absence of the membrane. Conversely, Fig. 10c,d illustrates the spectra of Ar plasma, once again in both the presence and absence of the membrane.

The strong atomic spectral lines (associated with N, N^+^) for the DC glow discharge N_2_ plasma are observed at 336.1, 374.8, 390.02, 404.7, 405.8, 426.4, 469.8, 651.6, 660.6, 676.9, 685.7, 724.2, 735.6, 746.8, 772.7, 868.6, 886.7 nm wavelength; whereas the weak molecular bands (associated with N_2_ and N_2_^+^) for N_2_ plasma are observed at 356.4 (N_2_^+^), 676.9 (N_2_^+^) and 759.1 (N_2_) nm wavelength.

Correspondingly, for Ar plasma treatment a wide number of strong atomic lines (Ar I and Ar II) are presented in the region of 600–1000 nm. The strong atomic lines are observed at 656.4, 695.8, 674.7, 695.8, 706.3, 726.6, 737.7, 749.7, 763.1, 772.04, 794.3, 800.9, 811.3, 826.08, 842.3, 851.8, 911.8 and 922.1 nm wavelength. In the spectral range spanning from 200 to 600 nm, numerous faint atomic lines of Argon II (Ar II) are detected. Specifically, lines are discerned at wavelengths of 418.8 nm, 426.5 nm, 433.4 nm, 460.5 nm, 476.4 nm, 485.6 nm, 547.8 nm, 549.2 nm, 561.6 nm, and 591.5 nm.

A noticeable reduction in the intensity of both atomic lines and molecular bands is distinctly observed in the presence of the membrane for both the N_2_ and Ar plasma. This observation suggests a depletion of plasma density in the presence of the membrane in plasma volume. During the plasma treatment, molecular species with low molecular weight are effectively removed from the membrane surface on basis on of the operating conditions^67^. For a constant electron temperature, the spectral line intensity of OES depends on the concentration of the excited species^72,74^. The concentration of the species released from the membrane surface due to plasma etching might not be quite enough to detect the spectral lines associated with the species via the existing spectrometer. Due to this reason, it is difficult to identify all the species, released from the membrane during N_2_ and Ar plasma treatment through OES. However, a faint spectral band related to the NH radical is identified at the wavelength of 379.3 nm in the presence of the membrane during N_2_ plasma treatment. Argon, in contrast, is characterized as an inert gas. Consequently, during Ar plasma treatment, it remains unreactive with the polymer species. Instead, it impinges upon the polymer surface, inducing the creation of vacancies by displacing loosely bound atoms and molecules through an etching process. The plasma treatment is conducted under high vacuum conditions, minimizing the probability of interactions with elements other than the process gas. Consequently, the activated surface does not undergo significant reactions during Ar plasma treatment. Thus, no discernible radicals are detected in the Optical Emission Spectroscopy (OES) spectra for Ar plasma treatment.

## Conclusion

A comprehensive investigation into the impact of N_2_ and Ar as plasma process gases on the enhancement of permeate flux in CN membranes during mud water treatment is presently underway. Plasma diagnostics utilizing Langmuir probe analysis indicate a slight decrease in plasma density in the presence of the membrane for both N_2_ and Ar plasmas, while electron temperature exhibits negligible changes. Extensive morphological analysis of plasma-treated and untreated membranes employing SEM, AFM, and BJH confirms increased pore size, macro void size, and etching effects with prolonged plasma exposure. Remarkably, Ar plasma demonstrates a superior morphological structure compared to N_2_ plasma. The formation of diverse hydrophilic groups following plasma treatment is substantiated through XPS analysis, while FTIR affirms the preservation of the membrane's bulk properties after plasma treatment. Optical Emission Spectroscopy (OES) analysis confirms the presence of various atomic and molecular species during plasma treatment, with a decrease in their intensity, compared to pure plasma, validating plasma-polymer interaction effects. Wettability studies based on water contact angle (CA) reveal a decrease in CA with increased plasma treatment time, signifying heightened hydrophilicity. Ar plasma exhibits superior hydrophilic characteristics compared to N_2_ plasma. The ageing behaviour of plasma-modified membranes is examined experimentally and a theoretical diffusion model is developed to validate the experimental results. Theoretical predictions align well with experimentally observed results under both plasma conditions. The present investigation indicates that the post-stabilization of the plasma-treated membrane takes place after 7 days for both vacuum and atmospheric medium. A slower rate of hydrophobic recovery is observed in vacuum mediums as compared to atmospheric mediums for both plasmas. From the gravity filtration process, it is observed that Ar plasma-treated membranes exhibit superior permeation characteristics compared to N_2_ plasma which might be due to the higher hydrophilicity and pore size of Ar plasma-treated membranes. To determine the most suitable Hermia model for describing fouling mechanisms, the Cake Filtration Model (n = 0) consistently emerges as the most fitting model for determining fouling mechanisms for untreated and plasma-treated CN membrane filtration. As a whole, Ar plasma treatment on CN membrane shows a better result in improving the hydrophilic nature of the membrane in comparison to N_2_ plasma treatment which in turn enhances the antifouling behaviour of the membrane for mud water treatment.

## Methodology

### Experimental assembly for plasma treatment

**Figure 11:**
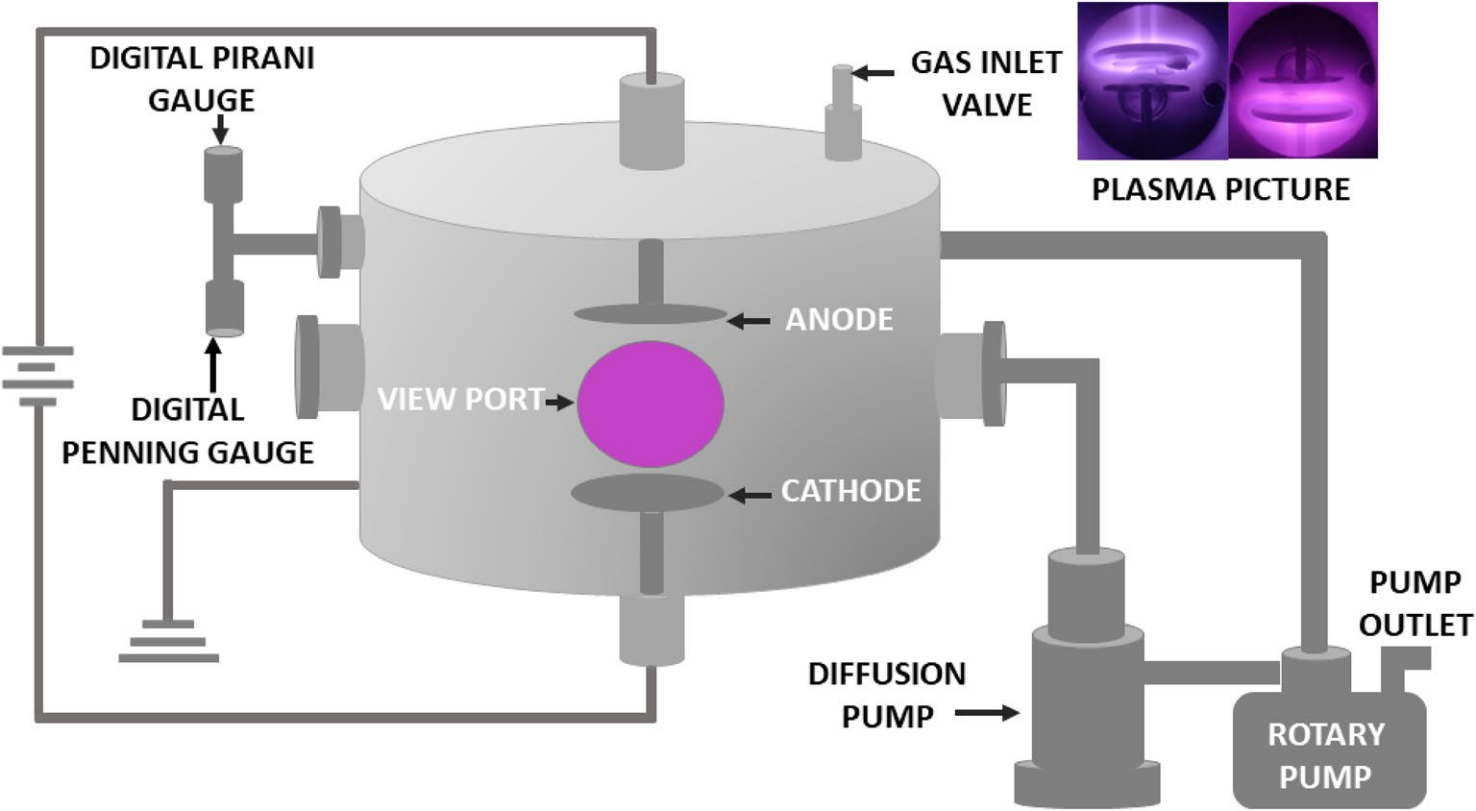
The schematic representation of plasma reactor.

The surface treatment of cellulose nitrate membranes is carried out in a glow discharge plasma reactor by producing N_2_ and Ar plasma. The schematic representation of the experimental assembly is shown in Fig. 11. The cylindrical cell is used as the plasma chamber. The volume of the chamber is ~ 3.5 × 10^3^ cm^3^ (Dia: 30.0 cm and height: 50.0 cm, made of SS-304L material). The chamber is evacuated through a diffusion pump with a pumping speed of 1000 L/min; aided by a rotary pump with a pumping speed of ~ 540 L/min. A base pressure of ~ 10^−6^ mbar is obtained with the pumping system. Two circular plates, made of SS-304L materials are used as electrodes to instigate the discharge with the help of a DC power supply (Make: Aplab, Rating: 1000 V/ 2.5A).

The operating pressure for plasma production is maintained at 2 × 10^−1^ mbar by feeding ultra-pure Argon or Nitrogen gas (99.99%) into the plasma chamber with the help of a digital flow controller (Model: ALICAT-MCN-100SCCM-D). The membranes are treated for 2 min, 7 min and 12 min, respectively for both the N_2_ and Ar plasma. To understand the role of process gases on the properties of the membrane, the discharge voltage and discharge current are maintained constant at 420 V and 20 mA for both the plasma in the present work. A cylindrical Langmuir probe is used to measure the plasma density (N_e_) and electron temperature (T_e_) of the plasma.

## Characterization techniques

### Wettability studies

In the current study, the water contact angles (CA) of both virgin and plasma-treated membranes are measured using the Sessile drop method. The measurement is carried out using an A-Cam contact angle analyser (Model: ADCAM-02). A 5 µL droplet of distilled water is placed, and the half-angle fitting method is utilized to obtain the static contact angle values. On the other hand, for precisely determine the wetting condition of the untreated and plasma-treated membrane, the advancing and receding contact angles are also investigated. To enhance accuracy, the average value derived from ten measurements for each set of samples is presented in the manuscript. All measurements are done at controlled temperature and humidity.

### Ageing studies

The study explores the ageing characteristics of plasma-treated membranes over a period of 30 days through contact angle measurements. To ascertain hydrophobic recovery, the water contact angle is measured at various time intervals after the plasma treatment utilizing the A-Cam contact angle analyser. A diffusion-based theoretical model is developed to verify the experimental findings in the recent work.

### Surface morphology

The surface morphology for both untreated and plasma-treated membranes is examined using Field Emission Scanning Electron Microscopy (FESEM; Carl ZEISS Gemini, Sigma 300, Germany). Prior to inspection, the samples are dried and a gold coating is applied in an Argon atmosphere. For clear imaging, the samples, acceleration voltage of 5.0 kV and magnifications of 10 kX and 30 kX are utilized and presented in the present work.

To assess surface roughness, Atomic Force Microscopy (AFM; Model NTEGRA Vita from NT-MDT) is employed on both the untreated and treated membranes. The roughness of the AFM images is analysed using GWYDDION software.

In the present experiment, the surface characterizations are carried out after the post-stabilization of the plasma-treated membrane; when the change in CA value with ageing time is insignificant.

### Surface chemistry

In the current study, X-ray photoelectron spectroscopy (XPS) (Model: ESCALAB Xi+; Make: Thermo Fisher Scientific Pvt. Ltd., UK) analysis is being conducted to investigate the chemical compositions of the pristine and plasma-treated membrane surface. The spectrometer is equipped with an Al K_α_ X-ray source operating at 1486.6 eV. Samples are exposed to monoenergetic soft X-rays with spot sizes ranging from 900 to 200 µm, and the emitted electrons undergo precise energy analysis. The base pressure within the analyser chamber is consistently maintained at approximately 5 × 10^−8^ mbar. This technique, known for its sensitivity, relies on the detection of subtle variations in binding energies for the real-time identification of the chemical state of the materials under investigation. Binding energy calibration is performed using the characteristic carbon line (C1s = 284.7 eV).

In the present work, Fourier transform infrared spectroscopy (FTIR) is conducted to identify the various functional groups present in both virgin and treated membranes. The IR spectra ranging from 400 to 4000 cm^−1^ are obtained using the compression technique with an Alpha Bruker II FTIR spectrometer and OPUS software.

### Pore characterization

The surface pore area distribution and pore diameter of untreated and plasma-treated membranes are investigated using the Barett–Joyner–Halenda (BJH) method at 77 K (Model: Quantachrome iQautosorb analyser, Anton-Paar, India)^24,75^.

### Antifouling test of untreated and plasma-treated membranes

The antifouling characteristics of both untreated and treated membranes are investigated using a gravity filtration process. The filtration rate of the membranes is measured under atmospheric pressure and at room temperature for mud water. The effective filtration area of the membrane is found to be approximately 16.0 cm^2^. The filtration cell is filled with mud water to measure the permeate flux, which is calculated for mud water using the following equation:

1 $$J_{W} = {\raise0.7ex\hbox{$Q$} \!\mathord{\left/ {\vphantom {Q {A \cdot \Delta t}}}\right.\kern-0pt} \!\lower0.7ex\hbox{${A \cdot \Delta t}$}}$$

where Q is the permeating water volume (L), A is the effective area of membrane (m^2^), Δt is the time of sampling (h) and J_W_ is the permeating flux (L/m^2^h)^24,76^.

The feed mud water is prepared in the laboratory using natural mud collected from the Deepor Beel of Assam, India. The samples are initially dried under sunlight. Then, the rocks, debris, and larger particles are removed from the dried mud samples. After this process, the samples are passed through a 20 µm sieve (ASTM-635) to remove further large particles. It is reported by Kalita et al.^77^ that the distribution of sand, clay, and silt in the mud samples, collected from Deepor Beel ranges from 47.2 to 53.0%, 23.9 to 31.2%, and 17.8 to 24.9%, respectively.

To prepare the mud water, 20 g of sludge (mud) is added to 1 L of distilled water and stirred using a magnetic stirrer for 30 min with a rotation speed of 400 rpm in the present work. After passing through a basic filtration process, the resulting mud water is then used as the feed solution for the filtration experiments.

### Percentage rejection rate of untreated and plasma-treated membranes

In the present work, the concentration of mud water in both the feed and permeate are determined using UV–spectrophotometer (model no. UV-1900I, SHIMADZU) at a wavelength of 309 nm. The percentage rejection rate of both the untreated and plasma-treated membrane are measured using the following formula

2 $$R = \left( {1 - C_{p}/C_{f} } \right) \times 100\%$$

where *C*_*p*_ and *C*_*f*_ are the permeate concentration and feed concentration^43,78–80^.

### Characterization of plasma

A cylindrical Langmuir probe with a diameter of 0.25 mm and a length of 10 mm is employed to measure the plasma density and electron temperature. Optical Emission Spectroscopy (OES), (Model: HR 4000, Make: Ocean Optics) is used to identify the spectral lines for both N_2_ and Ar plasma during the treatment of the membrane.

### Theoretical model for hydrophobic recovery

**Figure 12:**
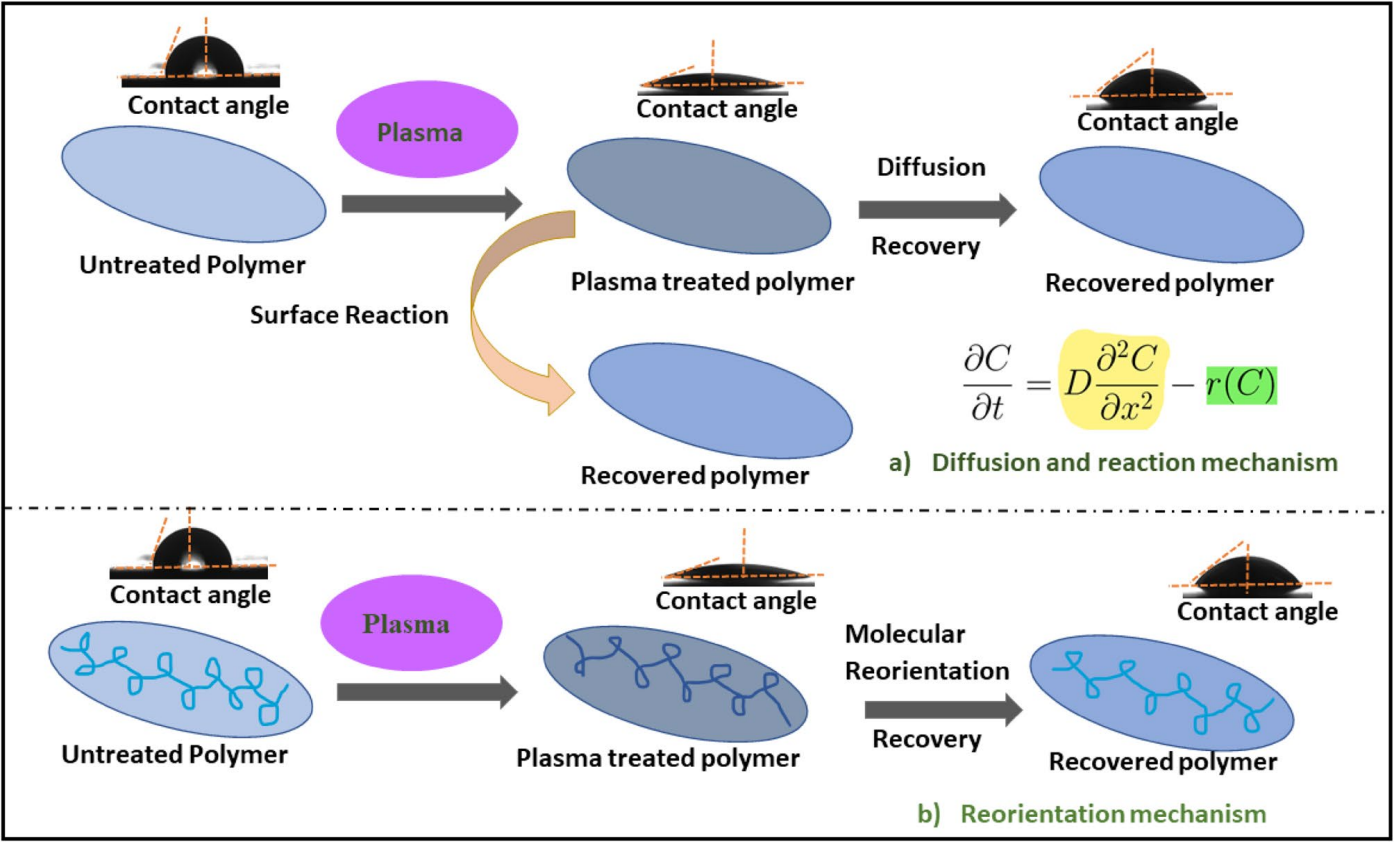
Mechanisms of hydrophobic recovery: (**a**) Diffusion and reaction mechanism and (**b**) Reorientation mechanism.

The hydrophobic recovery of the plasma-treated membranes is influenced by several factors, including treatment duration, humidity, gas type, storage conditions, and temperature. The time-dependent hydrophilicity behaviour of the plasma-treated membrane is due to the reorientation and diffusion of polar functional groups from the outermost layer into the bulk, as well as surface reactions. These mechanisms are commonly observed in the hydrophobic recovery process after plasma treatment^81^. Figure 12 illustrates the diffusion and surface reaction mechanisms involved in the recovery of hydrophobicity for plasma-modified polymers.

In the present work, a diffusion model is applied to verify the variation of contact angle with storage time at different plasma treatment conditions. The theoretical model is already discussed in the previous work^24^. A brief summary of the developed model is given below.

The diffusion and surface reaction of polar molecules during hydrophobic recovery can be written as:

3 $$\frac{{\partial C_{s} }}{\partial t} = D\frac{{\partial^{2} C_{s} }}{{\partial x^{2} }} - r$$

where the concentration of polar molecules is denoted by *C*, the Diffusion coefficient of the modified surface is defined as *D*, *x* is the distance from the top layer of the plasma-treated membrane, and *t* is the recovery time.

It has been assumed that the surface reaction follows 1st order reaction kinetics (Eq. 4). The variable separation method is used to solve the following equation

4 $$\frac{{\partial C_{s} }}{\partial t} = D\frac{{\partial^{2} C_{s} }}{{\partial x^{2} }} - kC_{s}$$

5 $$C_{s} \left( {x,t} \right) = X\left( x \right) T\left( t \right)$$

After solving the above equations, the final equations found as bellow,

6 $${\text{Cos}} \theta_{s} = {\text{Cos}} \theta_{U} + \left( {1 - {\text{Cos}} \theta_{U} } \right)\beta e^{{ - \left( {k + \lambda^{2} D} \right)t}}$$

where $\theta_{U}$ denotes the angle of contact angle of untreated membrane and $\theta_{s}$ denotes the contact angle of the membrane after an ageing period of *t* s.

The variation of the concentration and contact angle immediate after plasma modification on the topmost surface are as follows:

$$\begin{aligned} C_{s} \left( {0, 0} \right) & = \frac{{\left( {{\text{Cos}} \theta_{0} - {\text{Cos}} \theta_{U} } \right)}}{{\left( {1 - {\text{Cos}} \theta_{U} } \right)}} \\ \beta & = \frac{{\left( {{\text{Cos}} \theta_{0} - {\text{Cos}} \theta_{U} } \right)}}{{\left( {1 - {\text{Cos}} \theta_{U} } \right)}} \\ \end{aligned}$$

Equation 6 is used to estimate the hydrophobic recovery of plasma-treated membranes at different treatment condition. The contact angle data, measured experimentally are fitted with the theoretical results (Using Eq. 6) to study the effect of hydrophobic recovery for different storage medium of the plasma-treated membrane.

## Fouling model

When using a membrane to filter or separate substances over time, the flow rate (or flux) of the filtered substance might decrease. To identify the issues or factors that may affect the membrane's performance, it is necessary to examine the variation of flow rate with respect to time. Hermia et al.^82,83^ proposed an equation that is widely employed by researchers to characterize the batch filtration processes and identify the mode of flux decline as follows;

7 $$\frac{{d^{2} V}}{{dt^{2} }} = k\left( {\frac{dt}{{dV}}} \right)^{n}$$

where V is the cumulative flux at any time t. Here; k and n are system parameters. The value of n varies depending on the mode of filtration. Depending on the value of n, four different types of flux decline mechanisms can be derived from Eq. 7 which are discussed below;

### For n = 0, cake filtration model

In the model, it is assumed that the permeate flux decreases quadratically with time. This model is particularly relevant when a filter cake forms on the filtration medium. The Eq. 8 allows to estimate how the cake thickness evolves over time during the filtration processes.

8 $${\raise0.7ex\hbox{$1$} \!\mathord{\left/ {\vphantom {1 {J^{2} }}}\right.\kern-0pt} \!\lower0.7ex\hbox{${J^{2} }$}} = {\raise0.7ex\hbox{$1$} \!\mathord{\left/ {\vphantom {1 {J_{0}^{2} }}}\right.\kern-0pt} \!\lower0.7ex\hbox{${J_{0}^{2} }$}} + k_{d} t$$

### For n = 1, intermediate pore blocking model

The model suggests a linear relationship between the reciprocal of permeate flux and time. This relationship proves valuable when the filtration mechanism involves the gradual clogging of intermediate-sized pores within the filter medium. The theoretical equation for Intermediate Pore Blocking Model can be written as;

9 $${\raise0.7ex\hbox{$1$} \!\mathord{\left/ {\vphantom {1 J}}\right.\kern-0pt} \!\lower0.7ex\hbox{$J$}} = {\raise0.7ex\hbox{$1$} \!\mathord{\left/ {\vphantom {1 {J_{0} }}}\right.\kern-0pt} \!\lower0.7ex\hbox{${J_{0} }$}} + k_{d} t$$

### For n = 1.5, standard pore blocking model

The model is relevant when pore blocking occurs and the decrease in permeate flux follows a square root relationship with time. This model is often used to describe situations where the rate of flux decline is between that of the Intermediate and Complete Pore Blocking Models. The theoretical equation for Standard Pore Blocking Model can be expressed as;

10 $${\raise0.7ex\hbox{$1$} \!\mathord{\left/ {\vphantom {1 {\surd J}}}\right.\kern-0pt} \!\lower0.7ex\hbox{${\surd J}$}} = {\raise0.7ex\hbox{$1$} \!\mathord{\left/ {\vphantom {1 {\surd J_{0} }}}\right.\kern-0pt} \!\lower0.7ex\hbox{${\surd J_{0} }$}} + k_{d} t$$

### For n = 2, complete pore blocking model

The model describes scenarios where the permeate flux decreases exponentially with time due to the complete blocking of pores within the filter medium. The theoretical equation for Complete Pore Blocking Model can be expressed as;

11 $$\ln \left( {{\raise0.7ex\hbox{$1$} \!\mathord{\left/ {\vphantom {1 J}}\right.\kern-0pt} \!\lower0.7ex\hbox{$J$}}} \right) = \ln \left( {{\raise0.7ex\hbox{$1$} \!\mathord{\left/ {\vphantom {1 {J_{0} }}}\right.\kern-0pt} \!\lower0.7ex\hbox{${J_{0} }$}}} \right) + k_{d} t$$

In the above equations, J is the permeate flux at any time t, J_o_ is the initial permeate flux and k_d_ is the filtration coefficient. The unit of k_d_ changes depending on the value of n. These models are valuable for understanding different types of fouling processes.

## Data Availability

The data that support the findings of this study are available from the corresponding author upon reasonable request.
